# Phosphide of Zinc—A New Remedy

**Published:** 1868-12

**Authors:** 


### Phosphide of Zinc—A New Remedy.

The compounds of phosphorus offer many difficulties in their use as remedies, and these are often so decided as to render the therapeutical application of this element almost impossible, owing either to the obnoxious taste or unreliability, or both together. According to M. Vigier, of Paris, phosphide of zinc, however, unites in itself the characters of an excellent medicine, and appears well adapted to replace all other combinations of phosphorus. It is a grayish crystaline body, unchanged by the air, keeps well both in form of powder and of pills, and yet is readily decomposed in the stomach, with evolution of phosphureted hydrogen. This latter compound acts upon the animal organism precisely like the solution of phosphorus in oil; for it produces the same symptoms of poisoning, the same changes in the blood, local effusions of blood into the cellular tissue, and hemorrhages generally; also, congestion of the lungs, paralysis of the muscles of the heart, granular fatty degeneration of the cellular tissue in the liver and kidneys, etc.

Phosphide of zinc is produced by passing the vapors of phosphorus over zinc kept boiling in an atmosphere of hydrogen gas. It is thus obtained either crystaline, or spongy, or fused, but always of the composition P Z. 3. It is friable, of a vitreous fracture and a metallic lustre, is readily attacked by the acids, even by lactic acid, which will explain its ready assimilation in the stomach, and the generation of phosphureted hydrogen in the intestinal canal. Used as an injection, it acts equally but more slowly; and applied to the skin, only after the lapse of several days.

The experiments made with it show that the phosphorus retains but half its poisonous effect in the pure state. About $\frac{9}{10}$ of a grain, equal to nearly $\frac{1}{4}$ of phosphorus, is necessary for killing a rabbit of 6℔ in weight, the same effect being produced by half as much phosphorus in the form of *Ol. phosphoratum.* This would render 15 to 23 grains a toxical dose for man, and it may therefore be given with impunity, without unpleasant effects, except the alliaceous breath. The doses may be taken up to $\frac{6}{100}$ of a gr. per day. The following are modes of administration proposed by Vigier and Curiè:

*Pills of Phosphide of Zinc (Zinchi phosphorati.)*

| | | |
|---|---|---|
| ℞ Pulv. Zinchi phosphorati, | . . | grana xii. |
| " Glycyrrhizæ, | . . . . . | " iv. ss. |
| Syrupi Acaciæ, | . . . . | " xiii. ss. |

M. ft. pil. 100.

*Powder of Phosphide of Zinc.*

| | | |
|---|---|---|
| ℞ Zinchi phosphorati, | . . . | grana vi. |
| Amyli, | . . . . . . . . | " lxxv. |

M. ft. pulv. 50. —*Druggists' Circular.*

